# Foundations and principles of the Canadian living donor paired exchange program

**DOI:** 10.1186/2054-3581-1-6

**Published:** 2014-05-20

**Authors:** Shafi Malik, Edward Cole

**Affiliations:** Clinical Fellow Renal Transplantation Programme, Toronto General Hospital, 200 Elizabeth Street, Toronto, ON M5G 2C4 Canada; University Health Network, University of Toronto, 190 Elizabeth St, RFE 1S-409, Toronto, ON M5G 2C4 Canada

**Keywords:** Renal transplantation, Living donor exchange

## Abstract

**Purpose of review:**

Kidney paired donation (KPD) remains an important strategy to facilitate transplantation in patients who have a healthy and willing donor, but are unable to proceed with directed donation due to either ABO incompatibility or a positive cross-match against their intended donor.

**Sources of information:**

Personal knowledge, The Canadian Blood Services Database for Living Donor Exchange, published reports and personal communications.

**Findings:**

The national Living Donor Paired Exchange Programme (LDPE) in Canada was established in 2009. 235 transplants were completed of which 190 were registered recipients and 45 were from the deceased donor (DD) wait list. At 1 year, patient survival was 100%, graft survival 98%, with a biopsy proven acute rejection rate of 8%. The mean serum creatinine (Cr) at the end of one year was 109 mmol/l. Donor survival is 100%. Key to success are national standards for antibody testing and cross-matching, and for evaluating donors and recipients, as well infrastructure (software and personnel) to run the program. The structure of the Canadian program is compared with that of other programs in the United Kingdom, Australia, the Netherlands, and the United States.

**Limitations:**

This review does not include information on travel distances and difficulties, or patient satisfaction.

**Implications:**

National collaboration and acceptance of common standards is possible and leads to substantial benefits, especially for those patients who are hardest to match.

What was known before: Kidney paired donation is considered ethically acceptable. National and regional programs have been created in a number of countries.

What this paper adds: Key to the success of the Canadian national program are acceptance of standardized procedures and national and provincial support and oversight.

## Abrégé

**But de l’article:**

Les programmes d’échanges (PE) permettent de réaliser des transplantations à donneur vivant chez des receveurs qui ont des donneurs sains et volontaires mais qui sont incompatibles avec leur receveur en raison d’une incompatibilité de groupes sanguins ou immunologique.

**Sources d’informations:**

Connaissances personnelles, la base de données de la Société canadienne du sang pour les programmes par échanges de bénéficiaires, eécrits publiés et communications personnelles.

**Résultats:**

Le Registre de donneurs vivants jumelés par échanges de bénéficiaires a été inauguré en 2009. Jusqu’à maintenant, 235 transplantations ont été réalisées. Parmi les receveurs, 190 receveurs étaient inscrits dans le regsitre et 45 étaient en attente pour un donneur décédé. À un an, la survie du patient est de 100% et celle du greffon de 98%. L’incidence des rejet aigus prouvés par biopsie réanle est de 8%. La valeur moyenne de la créatinine sérique à un an est de 109 μmol/L. La survie des donneurs est de 100%. Les procédures normalisées nationales pour la détection d’anticorps, les épreuves de compatibilité croisée, l’évaluation des donneurs et des receveurs ainsi que les infrastructures (informatiques et ressources humaines) sont des éléments clés expliquant le succès et le fonctionnement du programme. Dans cet article, nous comparerons la structure du programme canadien avec d’autres programmes d’échange au Royaume-Uni, en Australie, aux Pays-Bas et aux Etats-Unis.

**Limites:**

Cet article de revue ne rapporte pas d’infomations sur les distances de voyagement, les difficultés ou la satisfaction des patients.

**Implications:**

Le programme d’échange canadien démontre qu’il est possible de collaborer nationalement et d’avoir un consensus sur des normes. Ceci permet de faire bénéficier nos patients en attente de greffe, particulièrement les patients qui sont difficiles à jumeler.

## Introduction

The results of renal transplantation have improved substantially. As well, various strategies have been employed to increase organ availability. Despite this, the number of patients on the waiting list continues to grow and demand for organs is ever increasing [1, 2]. The 2013 Canadian Organ Replacement (CORR) register estimated 40,385 people living with end stage renal disease (ESRD), 5,489 ESRD patients initiated renal replacement therapy (RRT) in 2011, whilst only 1,247 kidney transplants were performed during the same time period in Canada. At the end of 2011, there were 3,406 patients still waiting for a renal transplant, which is a 23% increase from 2005. As a result of this disparity, patients wait longer to receive a transplant while continuing on dialysis treatment and there is a mortality rate of 7.3 deaths per 100 patient-years on the waiting list [3].

**Figure 1:**
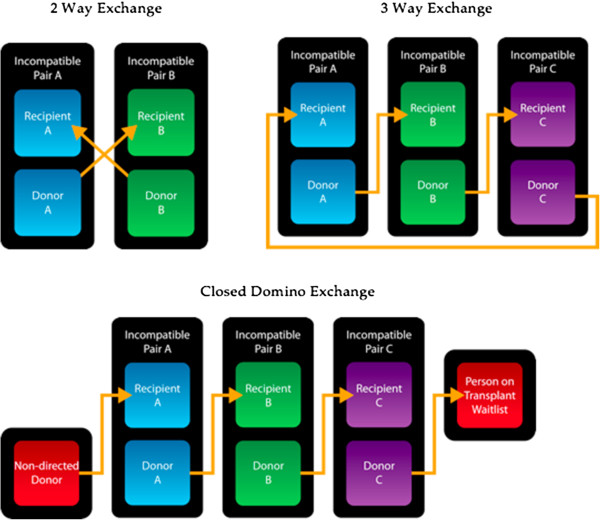
**Is a graphical representation of different types of exchange schemes.**

Living donor transplants provide a mortality benefit over deceased donor transplantation [1]. However, in order to receive a living donor transplant a willing, medically acceptable, and compatible donor is required. It is estimated that one third of patients with a willing, medically acceptable donor are unable to receive a transplant because of ABO incompatibility or the presence of a donor specific antibody (DSA) [2]. Kidney paired donation (KPD) is a strategy which aims to increase the number of living donor transplants by matching incompatible pairs. KPD was first proposed in 1986 by Felix Rapaport [4], but it wasn’t until 1997 that it was considered a valuable strategy to increase living donor transplants, when the ethics for such transplants were justified by LF Ross [5], and based on excellent results from living donor transplantation from unrelated donors (see Figure 1).

Segev et al have shown that the percentage of registry matches is higher if more pairs are entered [6]. As well, for difficult-to-match patients (those who are highly sensitized or blood group O recipients), participation in a larger program would increase their probability of finding a compatible donor because of larger donor pool size. These factors favour national registries over multiple independent registries, especially in countries with relatively small populations, like Canada.

### The Canadian programme

Until several years ago, based partly on provincial funding and organization of health care, there was no national transplantation collaboration or oversight. Canadian Blood Services (CBS) was ultimately entrusted by provincial and territorial governments to establish national organ donation and transplant registries. The first registry, the Canadian LDPE programme was established in 2009 by a collaboration between Canadian Blood Services and the Canadian renal transplant community. Collaboration and discussion with key leaders in the field of KPD played an important role in setting out the principles and foundations of the programme. Table 1 summarizes the key elements of the Canadian LDPE and Table 2 provides details on the matching algorithm.

**Table 1:** **Key elements of the LDPE program in Canada**

No.	Key elements
1	Standardization of HLA laboratory practice across all participating programmes.
2	Standardized work-up and acceptance criteria for living donors.
3	Match runs are performed centrally every four months using a protocol of optimization with a centralized software system developed by CBS.
4	National steering committee of transplant professionals to formulate and evaluate principles and to address logistical and medical issues promptly.
5	Dedicated central support staff at CBS to conduct and report on match runs.
6	Annual review of the allocation system to ensure equitable access to transplantation.
7	Donor travel to transplanting center with expense reimbursement by provinces.

**Table 2:** **The matching algorithm and points allotted for each attribute**

Attribute	Match points
Any transplant	100
Highly sensitized (cPRA ≥ 80%)	125
ABO Match: O to O	75
Paediatric recipient	75
Recipient is Prior Living Donor	75
HLA 0/6 Mismatch	75
Dialysis Wait Time (starting at initiation of dialysis)	Days/30
Geography: Same City	25
Donor/Recipient Age Δ ≤30 years	5
ABO Match: A to A, B to B, AB to AB	5
EBV Negative to Negative Match	5

**Figure 2:**
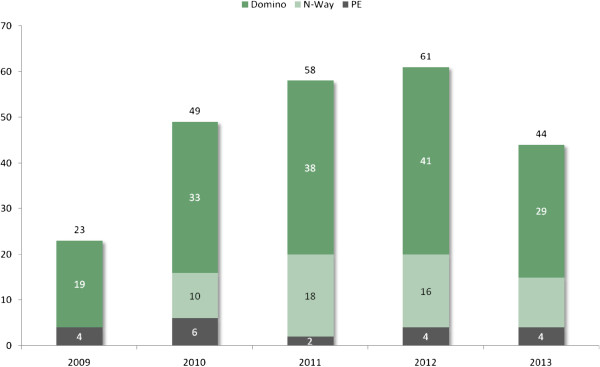
**Shows the number of transplants completed in the LDPE program by year and category.**

As of November 2013, 468 pairs were registered and a total of 58 non – directed donors (NDD) had been included. 235 transplants were completed of which 190 were registered recipients and 45 were from the deceased donor (DD) wait list. Of the 235 total transplants, 20 were paired exchanges or 2 way exchanges, 55 were from 16 multi way exchanges of 3, 4 or 5 chain lengths, and 160 resulted from domino exchanges; (46 dominos of 2-5 transplants each) (see Figure 2 - 2013 data partial). 259 pairs with a blood group O recipient have been registered and 86 (45%) of the 190 have undergone transplantation (see Table 3). Of these, 35 had a PRA of 1-79% and 36 a PRA of 80% or more. Table 4 provides an overview of key elements of paired exchange programmes worldwide.

**Table 3:** **The number of transplants for each blood group**

A to A	A to AB	AB to AB	B to B	O to A	O to B	O to O	Total
61	3	1	31	2	6	86	190
32%	2%	1%	16%	1%	3%	45%	100%

**Table 4:** **Comparison of key elements of KPD national programmes worldwide**

Country	Canada	UK	Australia	US	Netherlands
Name of programme	Living Donor Paired Exchange	Paired Living Kidney Donation	Paired Kidney Exchange Programme	Kidney Paired Donation	Living Donor Exchange Programme
Organization responsible	Canadian Blood Services	NHS Blood and Transplant	Organ and Tissue Authority	National program overseen by Organ Procurement and Transplant Network (OPTN) plus multiple independent registries	Dutch Transplant Foundation
Year national program established	2009 [7]	2007 [8]	2010 [9]	2010 [10, 11]	2004 [12]
Type of registry	National	National	National	National registry and smaller independent registries exist	National
Types of exchanges considered	Multi way and domino*	Multi way and domino	Multi way and domino	Multi way and domino	Multi way and domino
Bridge Donors	No	No	No	Yes	No
Desensitization programme in combination with KPD	No	No	Yes [13]	Yes [14]	No

*(Multi way exchanges can be 2 way, 3 way or more type of exchanges between pairs; domino exchanges are triggered by a non-directed altruistic donor which then sets of a series of transplants. When the last donor donates to the patient at the top of the deceased donor pool it is a closed domino; when the last donor does not donate but instead waits to set off another series of domino transplants this is called open domino exchange, and such donors are called bridge donors).

The average time taken for a chain to be completed by chain type is as follows: 101 days for paired exchange (PE), 129 days for multi way and 117 days for domino chains. 54% of matches have required travel. Sensitized patients with calculated panel reactive antibody (cPRA) <97% have had a 50% chance of a match within the program and sixty per cent of registered recipients with a cPRA between 80-96% have been transplanted. Transplant rates among the highly sensitized group of patients with cPRA >97% have been lower. 58% of proposed matches and 62% of proposed chains were completed. 55 chains collapsed due to 57 declined matches, of which, 6 were repaired and completed. 49 collapsed chains were not repaired (affecting 151 matched pairs). Of the 57 declined matches, 15(26%) were due to positive cross-matches, 24 were due to medical reasons, 14 due to non-medical reasons and finally 5 were as a result of surgical causes. In 2013, there were only 2 unexpected positive cross-matches.

The outcomes of KPD transplants are comparable to that of directed living donor transplants. At 1 year, patient survival was 100%, graft survival 98%, with a biopsy proven acute rejection rate of 8%. The mean serum creatinine (Cr) at the end of one year was 109 mmol/l. Donor survival is 100%.

### The United Kingdom programme

The KPD programme in the UK has been operating since 2007. Donors and recipients are assessed according to guidelines set by the British Transplantation Society/Renal Association [15]. Matching runs occur every 3 months. The matching algorithm uses a points-based system where points are allotted based on geographical proximity between pairs, calculated human leukocyte antigen antibody reaction frequency (cRF), HLA mismatch of potential transplant, and donor-donor age difference [8]. Initially, all potential two-way exchanges were identified with prioritization according to the points based system. Three-way exchanges were additionally considered after the first year. The initial results of the KPD programme were published in 2008. As of July 2008, 120 patients were registered in the KPD programme, of this, 8 proceeded to get a transplant. The reason given for this lower than expected transplant rate was the degree of sensitization in enrolled recipients (46% of the patients had a cPRA of >85%) [8]. Thus it may be, that easier to match patients were done locally without national involvement and only difficult to match patients were entered into the national registry. Individual centers were also found to be pursuing desensitization strategies for registered patients separate to the KPD programme. There is no requirement for donor to travel and kidneys are transported between centers. In the UK, NDDs have a choice to opt in to participate in a donor chain by being matched in a KPD run; otherwise the kidney is offered to a high priority patient on the DD wait list. High priority patients on the DD list take precedence (approximately 8% of altruistic donor kidneys are transplanted in a patient in the DD list); NDD kidneys are offered to these patients first, even if the NDD has opted to take part in the quarterly matching run. During the period 2012 -2013, 1068 patients received a living donor transplant, 76 transplants were from NDD donors and 55 paired living kidney donation transplants [16]. To date, 250 transplants have been completed, this includes 57 two way exchanges, 37 three way exchanges and 25 through altruistic donor chains where 25 patients at the top of the deceased donor wait list benefitted (R. Johnson, personal communication, January 6, 2014).

Changes were made to the system in 2012, where no priority was given to local exchanges, blood group compatible transplants were allowed, as compared to previously, where blood group identical transplants only were permitted. An incremental prioritization for recipients who remained unmatched in the scheme after each matching run was adopted. Further, compatible donor–recipient pairs could be enrolled and an extended matching criteria form for registering patients with low titer HLA and ABO blood group antibodies was introduced [17].

### The Australian programme

The first kidney paired donation (KPD) transplants were performed in Western Australia in 2007 and the national KPD programme was established in late 2010 as part of the Organ and Tissue Authority’s efforts to increase available organs from live donors [18]. Match runs are done every 3 months. The first run was performed in October 2010 and results from 9 match runs are currently available. The matching algorithm does not consider any HLA matching rules, and allocation is only based on acceptable mismatches by excluding donors from matching to recipients with DSAs greater than 2000 Mean Fluorescence Intensity (MFI). A computer algorithm helps select between competing match offers on the basis of pre-specified ranking rules, such as favouring 3-way over 2-way exchanges, in order to maximize the number of patients being transplanted, and then favouring patients with low versus high match probability, which primarily gives an advantage to a recipient with high versus low PRA [10].

As of October 2012, 115 pairs had registered in the national programme and 51 transplants were completed under the auspices of the national programme (A total of 152 pairs had registered by 30 June 2013, which includes pairs registered prior to establishment of a national program. A total of 72 transplants have been completed).

In the national programme, 63% of enrolled pairs in the first 2 years found a match with 46% of them receiving a transplant. 33% of registered candidates had a cPRA >95%. 42% of transplanted patients had a cPRA >75% and mean cPRA was 55%. Blood group O recipients are often considered to be disadvantaged in a KPD program as they can only receive an organ from a blood group compatible donor. In the Australian KPD program, 56% of transplant recipients were blood group O, this has been possible due to ABO desensitization being included as part of KPD [19]. ABO-incompatible donors were accepted for 36 patients and of these, 10 recipients successfully underwent transplantation following desensitization [13].

### The Dutch programme

Eight year outcomes of the Dutch KPD programme were published in 2011 [12]. All transplants performed since programme inception in 2004 to 2011 were analysed. 472 pairs were enrolled, consisting of 269 due to ABO blood type incompatibility and 203 due to positive cross match with their intended donor. 187 transplants were performed during this period, which is 40% of registered candidates. Of the 187 transplants completed, 83 were ABO incompatible and 104 had a positive cross match against their intended donor. The 5-year uncensored graft survival was 85% and the death censored graft survival was 89%.

### The US programme

In the US, multiple independent registries exist in addition to a national registry administered by UNOS. KPD may be underutilized in the US and factors contributing to underutilization include insurance costs, reimbursement to donor when there are different insurance providers, and fragmented registries [20]. The KPD program at Methodist Specialty and Transplant Hospital, San Antonio, runs a successful independent registry. The program reported its results in 2012, over a 3-year period, a total of 134 paired donor transplants were performed, including 117 incompatible pairs and 17 compatible pairs. Transplants included 2 way, 3 way exchanges and 3 chains initiated by an NDD, 44% of patients transplanted had a PRA of >80% [21]. Data from UNOS for the period between 2000 – 2007 showed that 209 patients underwent transplantation through KPD, no differences in survival were found when compared to matched live directed donation controls performed during the same period [22].

## Review

The national LDPE programme in Canada has been a rewarding success story. It is important to appreciate that the benefit of LDPE is not only that more patients get living donor transplants with the associated benefits, but also that all of those patients, who would previously have been on deceased donor lists, no longer are on them. This reduces waiting time for those without a living donor. The success of the programme is related to national collaboration, standardized antibody testing and standardization of both the workup and acceptance criteria for donors, centralized allocation software, operational oversight, dedicated central support staff, and government support of logistics for organization and travel.

The Canadian national LDPE program was established later than similar programs in the UK and Netherlands, but has seen good success in a relatively short span of time. As of November 2013, 235 transplants were completed which is higher than the reported transplants in other national programmes worldwide. The relatively small geographic area in the Netherlands enables them to do cross matches in a central laboratory. Other national programs are yet to standardize HLA laboratory techniques. The publicly funded healthcare system in Canada has obviated the argument of financial responsibility unlike in the US where there is ongoing debate on establishing a standard acquisition charge for KPD by private insurers [23]. A high number of proposed matches have been completed and the average time taken to complete a chain has also been low, for example in the Australian KPD programme, 12% of patients have had to wait in excess of four months to receive their transplant and 8% of proposed matches did not proceed due to a subsequent unexpected positive cross match within the same programme. In Canada, only 2 such unexpected positive cross matches have occurred in the last year. Chain collapse results in waste of resources and is disappointing to patients. Standardization of HLA laboratory practices and standardized acceptance criteria for donors has helped in minimizing chain collapses in our programme.

There are important considerations for the future. Candidates with cPRA > 97% have a lower transplant rate than other highly sensitized patients. Strategies to improve this, including the national registry for highly sensitized patients, are under active consideration. For instance, consideration of desensitization in association with LDPE is suggested by some early encouraging results of ABO and low level DSA desensitization from programs in Australia and the US [13, 14]. The future shipment of kidneys has the potential to reduce cost and simplify logistics. It is now known that living donor kidneys can withstand longer cold ischemia time with no deleterious effects on outcomes [24, 25]. It is clear from the available data that participation across Canada varies substantially by centre. We believe that LDPE is an important way of increasing access to transplantation and encourage all centers to consider it for every case with a medically suitable but incompatible donor.

## Conclusion

The Canadian LDPE programme is an example of success achieved by national integration and collaboration. Hopefully, the success to date will emphasize the benefits of LDPE to both patients and centers, resulting in a further increase in pairs entered, matches and transplants.

## Abbreviations

CBS: Canadian blood services
CORR: Canadian organ replacement register
cRF: Calculated human leukocyte antigen antibody reaction frequency
DD: Deceased donor
DSA: Donor specific antibody
EBV: Epstein–barr virus
ESRD: End stage renal disease
HLA: Human leukocyte antigen
KPD: Kidney paired donation
LDPE: Living donor exchange programme
MFI: Mean fluorescence intensity
NDD: Non-directed donor
OPTN: Organ procurement and transplant network
PRA: Plasma renin activity
RRT: Renal replacement therapy
UNOS: United network for organ sharing.
